# Diagnosis of Listeria monocytogenes Meningitis Using the FilmArray Meningitis/Encephalitis Panel in a Patient With Prior Antibiotic Exposure: A Case Report

**DOI:** 10.7759/cureus.87687

**Published:** 2025-07-10

**Authors:** Yu Miyazaki, Koki Tominaga, Takuya Adachi

**Affiliations:** 1 Department of Infectious Diseases, Tokyo Metropolitan Toshima Hospital, Tokyo, JPN; 2 Department of Infectious Diseases, Tokyo Metropolitan Cancer and Infectious Diseases Center Komagome Hospital, Tokyo, JPN

**Keywords:** bacterial meningitis, filmarray, infectious disease medicine, listeria monocytogenes, multiplex pcr

## Abstract

Bacterial meningitis is a medical emergency with high mortality rates and a significant risk of complications. Conventional diagnostic methods, such as Gram staining, sometimes fail to identify the causative pathogen, and cultures take time to yield results. The FilmArray meningitis/encephalitis (ME) Panel, a molecular diagnostic tool, identifies 14 pathogens in approximately one hour. We report a case of *Listeria monocytogenes* meningitis in a 79-year-old man, diagnosed using the FilmArray ME Panel after antibiotic exposure. The diagnosis was made on day seven, three days after admission, using a cerebrospinal fluid sample that had been stored due to the unavailability of the test over the weekend. Despite negative cultures, the panel facilitated diagnosis and effective treatment with ampicillin and gentamicin, leading to full recovery without sequelae. This case highlights the utility of the FilmArray ME Panel in challenging diagnostic scenarios and underscores the importance of ensuring its availability during after-hours and weekends to facilitate timely care in critical conditions.

## Introduction

Bacterial meningitis is a medical emergency characterized by high mortality rates and a significant risk of long-term complications [1]. Among the various pathogens, *Listeria monocytogenes* is a relatively uncommon but important pathogen, especially in elderly and immunocompromised patients. It is typically acquired through contaminated food, such as unpasteurized dairy or processed meats, and accounts for approximately 9% of bacterial meningitis cases in individuals over 50 years of age [2]. Traditionally, the diagnosis of bacterial meningitis relies on Gram staining and culture. However, Gram staining sometimes fails to identify the causative pathogen, and culture takes time to yield results. Furthermore, the sensitivity of cerebrospinal fluid (CSF) Gram stain and culture is markedly reduced in patients who have received prior antibiotic therapy [3]. Recently, molecular diagnostic tools that allow a syndromic approach to meningitis/encephalitis (ME) diagnosis have become available. Since results can be obtained in about one hour, these methods have generated significant interest [4]. However, the FilmArray ME Panel is still a relatively new diagnostic tool, and there have been few reports of its successful use in diagnosing *L. monocytogenes* meningitis following antibiotic exposure. Here, we present a case of* L. monocytogenes *meningitis in an elderly patient that was successfully diagnosed using the FilmArray ME Panel after the initiation of antibiotic treatment. The case highlights the importance of early molecular testing in cases of suspected bacterial meningitis and the need for improved accessibility of such diagnostics, particularly during off-hours.

## Case presentation

A 79-year-old man with hypertension had traveled to Vietnam from nine days to two days before the onset of symptoms. The patient presented to a nearby hospital with a two-day fever and a one-day history of altered consciousness. His blood and urine tests and computed tomography (CT) scans of the head, chest, and pelvis were unremarkable. He was admitted to the hospital for further evaluation of the fever, and ceftriaxone was initiated. However, his fever and altered consciousness worsened. To broaden antimicrobial coverage for other possible pathogens, particularly Gram-negative rods and anaerobes, ceftriaxone was switched to piperacillin-tazobactam. He was transferred to our hospital on day four. There was no history of consuming cheese, cured meats such as prosciutto, or similar foods. Additionally, there was no family history of tuberculosis.

**Figure 1:**
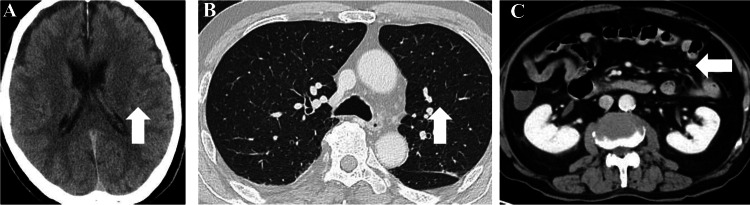
CT scans on illness day four. (A) Non-contrast CT scan of the head. (B) Contrast-enhanced CT scan of the chest. (C) Contrast-enhanced CT scan of the abdomen and pelvis. No significant abnormalities were observed in any of the scans. The arrows indicate typical structures to show the absence of pathological changes.

On arrival, the patient exhibited altered consciousness (Glasgow Coma Scale: E4V4M6) but was afebrile and hemodynamically stable. No significant abnormalities were noted on physical examination. Rapid antigen tests for malaria and dengue fever were negative. CT scans of the head, chest, and pelvis showed no abnormalities (Figures 1A-1C). Blood and urine cultures were also submitted at our hospital, and piperacillin-tazobactam (4.5 g) was continued. Due to progressive neurological deterioration overnight, the antibiotic was switched to meropenem 1 g, and empirical minocycline 200 mg was added. Although there was no clear episode of insect bites, rickettsial infection was considered a possible differential diagnosis, given the patient's recent travel to Vietnam, where rickettsial diseases are endemic. The lack of clinical response to prior antibiotics raised concern for pathogens not adequately covered, including Rickettsia species. Therefore, minocycline was added to cover possible rickettsial infections.

**Figure 2:**
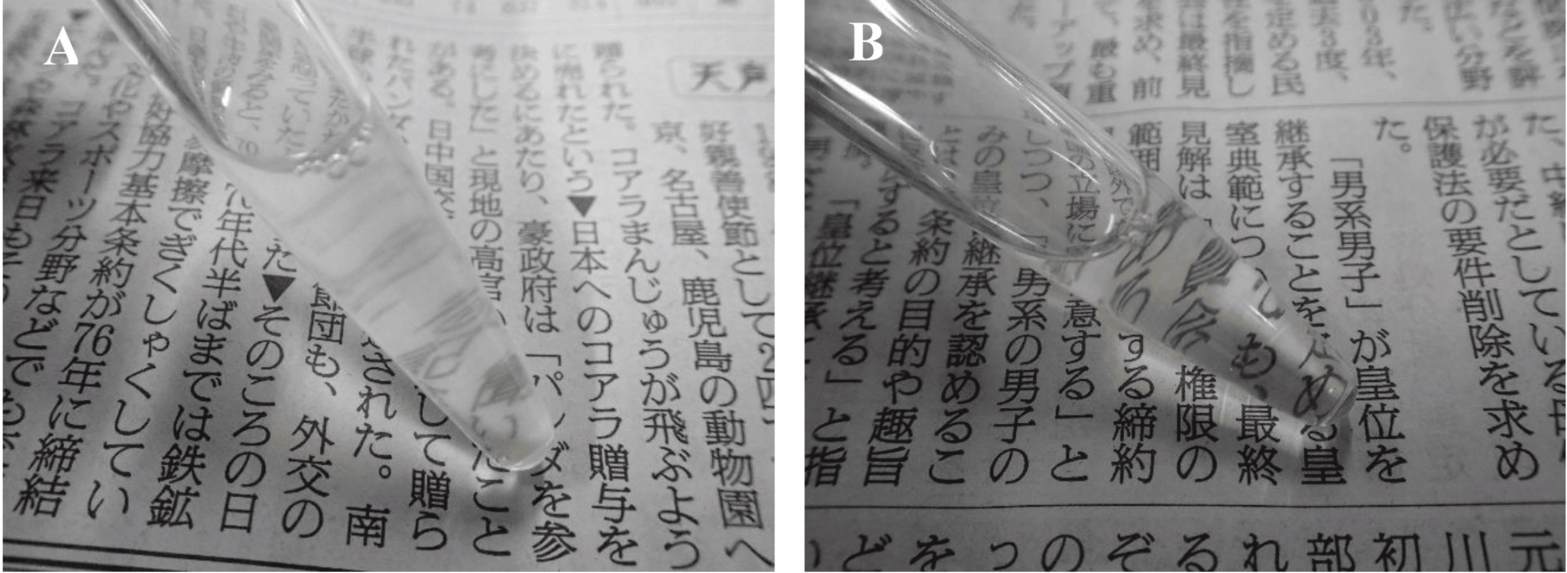
Cerebrospinal fluid findings in Listeria monocytogenes meningitis. (A) Turbid cerebrospinal fluid observed on illness day five. (B) Slightly turbid cerebrospinal fluid on illness day nine, showing signs of clearing.

On illness day five, the patient's consciousness deteriorated to E3V3M3, and neck stiffness was noted. The blood test showed elevated inflammatory markers, with a white blood cell (WBC) count of 16,100 /μL, C-reactive protein (CRP) level of 40.9 mg/dL, and a glucose level of 280 mg/dL (Table 1). The CSF obtained by lumbar puncture (LP) was turbid (Figure 2A), with an initial pressure of 370 mmH_2_O, WBC of 3,237/μL (82% neutrophils) (reference range: 0-5/μL), a decreased glucose level (101 mg/dL in CSF), and protein levels of 407 mg/dL (reference range: 10-40 mg/dL) (Table 2). However, the Gram stain was negative. The negative Gram stain may have resulted from prior antibiotic treatment, which reduces bacterial load and impairs the sensitivity of conventional microbiological tests.

**Table 1:** Blood test.

Variables	On illness day 5	Day 9	Day 28	Reference range	Unit
White blood cell	16,100	9,400	6,300	3,300-8,600	/μL
Neutrophils	87.6	93.2	16.0	37-80	%
Hemoglobin	14.0	13.1	10.5	11.6-14.8	g/dL
Hematocrit	40.1	39.2	30.2	35.1-44.4	%
Platelet	18.1	24.2	27.4	15.8-34.8	×10^4^/μL
Aspartate aminotransferase	34	62	36	13-30	U/L
Alanine aminotransferase	24	62	100	7-23	U/L
Lactate dehydrogenase	331	378	252	124-222	U/L
Total bilirubin	0.9	0.8	0.5	0.4-1.5	mg/dL
Total protein	6.7	-	5.9	6.6-8.1	g/dL
Albumin	3.0	2.2	2.6	4.1-5.1	g/dL
Creatine kinase	360	344	17	41-153	U/L
Blood urea nitrogen	14.4	25.9	9.0	8-20	mg/dL
Creatinine	1.2	0.85	0.83	0.46-0.79	mg/dL
Sodium	140	155	141	138-145	mmol/L
Potassium	2.7	4.0	3.8	3.6-4.8	mmol/L
Chloride	100	112	106	101-108	mmol/L
Glucose	280	-	108	65-109	mg/dL
C-reactive protein	40.9	7.7	0.27	0.00-0.14	mg/dL

**Table 2:** Cerebrospinal fluid analysis.

Variables	On illness day 5	Day 9	Day 28	Reference range	Unit
Nucleated cells	3,237	498	18	<5	/μL
Neutrophils	2,645	4	0	<5	/μL
Lymphocytes	592	494	18	<5	/μL
Glucose	101	207	60	55-75	mg/dL
Protein	407	460	109	10-40	mg/dL

Based on these findings, bacterial meningitis was suspected, and treatment with meropenem 2 g every eight hours and dexamethasone 9.9 mg every six hours was initiated. As the FilmArray ME Panel test could not be performed due to the weekend, the cerebrospinal fluid sample was stored for later analysis. On day seven, stored CSF tested positive for *L. monocytogenes* using the FilmArray ME Panel. This multiplex PCR assay provides results in approximately one hour and demonstrates high analytical sensitivity, with a lower limit of detection around 10³ CFU/mL for most bacterial targets. In comparison, conventional Gram stain and culture typically require higher bacterial loads - up to 10⁵ CFU/mL - and take 24-72 hours to yield results. While the FilmArray ME Panel shows sensitivities of 85% to nearly 100% depending on the pathogen, data for *L. monocytogenes* remain limited due to its rarity of positive cases in validation studies. The diagnosis of *L. monocytogenes* meningitis was made, and the antibiotic regimen was changed to intravenous ampicillin (2 g every four hours) and gentamicin (120 mg every eight hours). On the same day, the patient developed seizures. A consultation with the neurologist was made, and levetiracetam was initiated. However, as the seizures persisted, the patient was transferred to the intensive care unit. Fosphenytoin sodium and midazolam were added, after which the seizures resolved. On day nine, blood tests showed improvement in inflammatory markers (Table 1). A repeat LP performed on the same day revealed slightly turbid cerebrospinal fluid, with improved clarity compared to the initial CSF (Figure 2B). The results showed a WBC of 498/μL, normal glucose, and protein levels of 460 mg/dL (Table 2), demonstrating a favorable response to the targeted therapy. Consequently, gentamicin was discontinued. In addition, no other organisms were detected in the CSF cultures taken on day five, and dexamethasone was discontinued.

**Figure 3:**
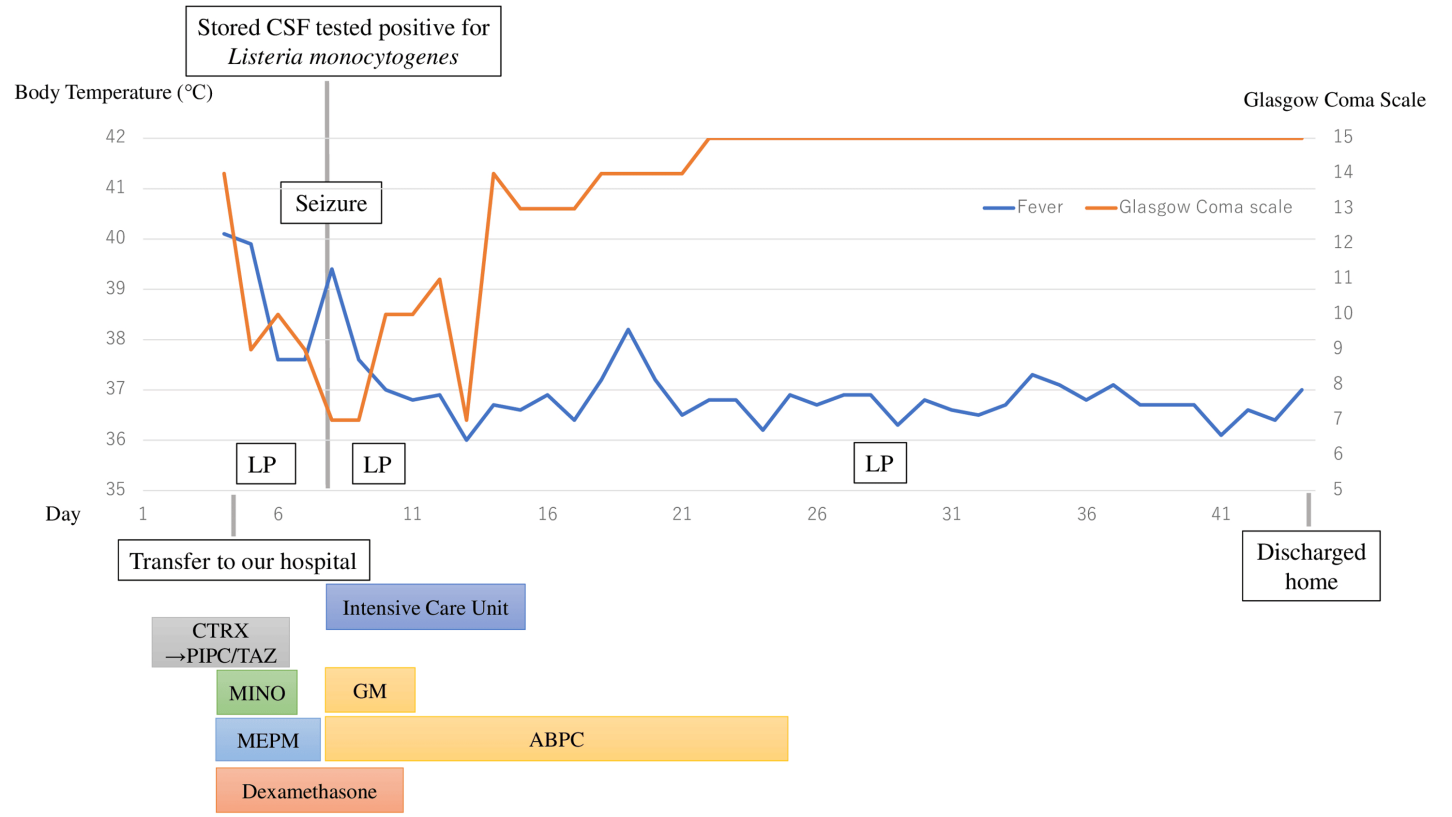
Clinical timeline of the patient with Listeria monocytogenes meningitis. This figure summarizes the key clinical events, antimicrobial treatments, diagnostic procedures, and neurological complications. Empiric therapy was escalated from ceftriaxone (CTRX) to piperacillin-tazobactam (PIPC/TAZ) and subsequently to meropenem (MEPM) and minocycline (MINO) due to worsening consciousness. A lumbar puncture (LP) performed on illness day five raised suspicion for bacterial meningitis, prompting an increased dose of MEPM and the initiation of dexamethasone (DEX). As the FilmArray meningitis/encephalitis (ME) Panel could not be performed over the weekend, the cerebrospinal fluid sample was stored. On illness day seven, *Listeria monocytogenes* was identified by the ME panel, and targeted therapy with ampicillin (ABPC) and gentamicin (GM) was initiated. The patient experienced seizures on the day of diagnosis and was admitted to the intensive care unit (ICU). By illness day nine, clinical and laboratory improvement was observed, and both GM and DEX were discontinued. The patient was discharged home without neurological sequelae on day 44.

The fever and altered consciousness gradually improved. Fosphenytoin sodium and midazolam were discontinued, and oral levetiracetam was continued. On day 28, blood tests showed that inflammatory markers had nearly normalized (Table 1). CSF analysis revealed a WBC of 18/μL, normal glucose levels, and a protein level of 109 mg/dL (Table 2). This progressive normalization of CSF parameters correlated with the patient's clinical recovery. CSF cultures at our hospital and blood cultures from both hospitals did not yield any bacteria. CSF acid-fast bacilli culture and tuberculosis PCR were negative. The patient was treated with intravenous ampicillin for a total of three weeks, gentamicin for four days, and dexamethasone for four days for bacterial meningitis. The patient was discharged home on day 44 (Figure 3). Upon discharge, the patient's Hasegawa Dementia Scale-Revised score was 25/30, with no evidence of cognitive dysfunction related to the illness.

## Discussion

We report a case of *L. monocytogenes* meningitis diagnosed using the FilmArray ME Panel in a patient with prior antibiotic exposure who fully recovered without any sequelae. This case highlights two key issues: first, the diagnostic value of the FilmArray ME Panel in patients with suspected bacterial meningitis who have already received antibiotics; and second, consistent access to the panel, including during weekends and nighttime hours, is crucial. In addition, this case underscores the importance of implementing hospital-wide protocols to ensure 24/7 accessibility to molecular diagnostics in critical care settings. Such policies could help prevent diagnostic delays and improve clinical outcomes.

*L. monocytogenes* is a Gram-positive bacillus and facultative intracellular bacterium, commonly present in the environment, including soil, vegetation, and animal reservoirs [5]. Listeriosis is considered one of the most common bacterial foodborne infections and is frequently transmitted through contaminated foods, such as dairy products and processed meats. The incubation period is approximately eight days (range: 1-67 days) [6]. While immunocompromised individuals, including cancer patients and those with HIV, are at high risk of infection, meningitis and sepsis can also occur in children and elderly individuals without underlying conditions. *L. monocytogenes* is responsible for approximately 9% of bacterial meningitis cases in individuals over 50 years of age and is a key pathogen in bacterial meningitis [2]. When neurological involvement occurs, the fatality rate can be as high as 30% [7]. Therefore, timely diagnosis and appropriate treatment of *L. monocytogenes* meningitis are critically important. However, antibiotic pretreatment significantly reduces the sensitivity of the CSF Gram stain from 25.7% to 13.4%, and CSF culture from 66.1% to 48.5% [3], thereby making the diagnosis challenging.

Recently, the FilmArray ME Panel has gained attention for its ability to identify 14 common agents of community-acquired ME in about one hour. These agents include *Escherichia coli* K1, *Haemophilus influenzae*,* L. monocytogenes*, *Neisseria meningitidis*, *Streptococcus agalactiae*, *Streptococcus pneumoniae*, cytomegalovirus, enterovirus, herpes simplex virus 1 (HSV-1), HSV-2, human herpesvirus 6, human parechovirus, varicella-zoster virus, and *Cryptococcus neoformans*/*Cryptococcus gattii*.

Compared to conventional diagnostic methods, the FilmArray ME Panel has been shown to reduce the time to diagnosis of ME, the duration of antibiotic therapy, and the length of hospital stay [8]. However, there are no reported cases of its use in diagnosing patients who have received prior antibiotic treatment. In this case, the patient developed *L. monocytogenes* meningitis after traveling to Vietnam; however, there was no suspicious dietary history, and the source of infection or its connection to the trip remains unclear. Due to prior antibiotic exposure, cerebrospinal fluid cultures were negative. However, the FilmArray ME Panel enabled the diagnosis of *L. monocytogenes* meningitis, leading to a life-saving outcome without any neurological sequelae.

The impact of antibiotic treatment on the test performance of the FilmArray ME Panel has not been evaluated. In patients with prior antibiotic exposure, the sensitivity of CSF Gram staining typically requires a microbial load of 10⁵ CFU/mL or higher, whereas the FilmArray ME Panel has a minimum detection sensitivity of 10³ CFU/mL or higher [9]. This suggests that, in this patient, the microbial load in the CSF was below the detection threshold of Gram staining but above the detection threshold of the FilmArray ME Panel. These findings suggest that the FilmArray ME Panel may aid in diagnosing bacterial meningitis, even in patients with prior antibiotic exposure.

This case highlights the utility of the FilmArray ME Panel. However, at our hospital, the FilmArray ME Panel is performed by laboratory technicians in the microbiology department. Since microbiology technicians are unavailable during nighttime hours and weekends, and all laboratory technicians rotate shifts regardless of specialty, the FilmArray ME Panel could not be utilized during these times. In our case, CSF was collected over the weekend, preventing the use of the FilmArray ME Panel at that time. According to the manufacturer’s instructions, specimens may be stored at room temperature (approximately 23°C) for up to one day or refrigerated (approximately 4°C) for up to seven days [9]. In this case, the specimen was stored at 3°C for two days before undergoing analysis with the FilmArray ME Panel. As a result, the FilmArray ME Panel could not be performed on the same day, delaying the diagnosis of *L. monocytogenes* meningitis. Institutional policies and resource allocation should prioritize the establishment of around-the-clock availability of such molecular diagnostic platforms, particularly for patients in emergency or intensive care settings.

Despite its advantages, the FilmArray ME Panel has limitations. First, the impact of prior antibiotic treatment on the panel’s performance has not been fully evaluated. Second, although no false positives for *L. monocytogenes* have been reported in previous studies, the organism was not detected at all, making it impossible to assess the test’s sensitivity for this particular pathogen [4]. Therefore, test results should always be interpreted in conjunction with the clinical context and other laboratory findings. A cautious and integrated diagnostic approach is essential to avoid misdiagnosis or delayed treatment.

## Conclusions

This case illustrates the successful diagnosis of* L. monocytogenes* meningitis using the FilmArray ME Panel in a patient with prior antibiotic exposure. The patient made a full recovery without any neurological sequelae, highlighting the clinical value of early molecular diagnostics in suspected cases of bacterial meningitis, particularly when conventional methods may be unreliable.

To ensure timely diagnosis and optimal outcomes in life-threatening central nervous system infections, it is essential that healthcare systems implement protocols and allocate resources to provide 24/7 access to molecular diagnostics, including during nights and weekends. Broader adoption of such tools and infrastructure could enhance the speed and accuracy of meningitis diagnosis, especially in critical care settings.
